# Risk factors for acute injuries and overuse syndromes of the shoulder in amateur triathletes - A retrospective analysis

**DOI:** 10.1371/journal.pone.0198168

**Published:** 2018-06-01

**Authors:** Dominik Schorn, Tim Vogler, Georg Gosheger, Kristian Schneider, Sebastian Klingebiel, Carolin Rickert, Dimosthenis Andreou, Dennis Liem

**Affiliations:** Department of General Orthopedics and Tumor Orthopedics, Münster University Hospital, Münster, Germany; University of Rome, ITALY

## Abstract

**Objectives:**

To investigate the prevalence of shoulder-related acute and overuse injuries in triathletes and examine the role of possible risk factors, in order to identify potential preventive measures.

**Methods:**

We performed a retrospective epidemiologic study of 193 amateur triathletes between June and August 2013 and evaluated their competition and training habits, as well as the presence of acute and overuse injuries of the shoulder sustained during the past 12 months. Contingency tables were analyzed using Pearson’s chi-squared test. Normally distributed data were compared with the independent samples t-test, while non-parametric analyses were performed with the Mann-Whitney U test. Binary logistic regression was used to identify important predictors of injuries.

**Results:**

12 participants (6%) sustained acute injuries and 36 athletes experienced an overuse injury. The acute injury rate amounted to 0.11 per 1000 hours of training and the overuse injury rate to 0.33 per 1000 hours of training. There was no association between athletes’ age, height, weight, BMI, a history of shoulder complaints or triathlon experience in years and acute or overuse injuries. Male athletes had a trend for sustaining more acute injuries then female athletes (8% vs. 2%, p = 0.079). Athletes with acute injuries spent a significantly higher amount of time per week doing weight training (p = 0.007) and had a trend for a higher weekly duration of cycling training (p = 0.088). Athletes with overuse injuries participated in a significantly higher number of races compared to athletes without overuse injuries (p = 0.005). The regular use of paddles was associated with a significantly higher rate of overuse injuries (24% vs. 10%, p = 0.014).

**Conclusion:**

The regular use of paddles during swimming training appears to be a risk factor for the development of overuse injuries, while an increased duration of weight and cycling training seems to be associated with a higher rate of acute injuries.

## Introduction

Triathlon is a multidisciplinary endurance sport that combines swimming, cycling and running.[1] Since its inception in the 1970s it has gained in popularity across the globe among both recreational and professional athletes, and it became an Olympic sport in 2000.[1, 2] Triathlon competitions are usually run over four race distances; the shortest distance is the Sprint (750 m swim, 20 km bicycle, 5 km run), followed by the Olympic (1500 m swim, 40 km bicycle, 10 km run), Long (3000 m swim, 80 km bicycle, 20 km run) and Ironman (3800 m swim, 180 km bicycle, 42.2 km run).[1, 3]

While the multidisciplinary nature of triathlon promotes strengthening of the entire body and the effects of triathlon have been shown to be more than the sum of its component sports,[4] the high training volume and the intense physical stress in competition place high demands on the athletes’ bodies.[5] As a result, sport-related acute and overuse injuries are common in triathletes, with a reported prevalence of 37–91%.[1, 3, 6, 7] The most common sites of injury identified in triathletes are the lower limb, the back and the shoulder.[1, 3] However, while the role of a number of intrinsic (internal factors, inherent to the athlete) and extrinsic (external factors, independent of the athlete) risk factors in triathlon-related injuries has been evaluated in the literature, the reported findings have been conflicting, especially regarding the influence of anthropometric parameters, weekly training load and triathlon experience.[2, 3] Furthermore, no studies, to our knowledge, have evaluated in depth the factors associated with acute and overuse injuries of the shoulder caused by triathlon training.

The goal of this epidemiological study was to investigate the prevalence of shoulder-related acute and overuse injuries in triathletes. We also aimed at examining the role of possible risk factors, in order to identify potential preventive measures.

## Materials and methods

### Study design

Between June and August 2013 triathletes training in local triathlon clubs, competing in local triathlon events or visiting German triathlon-related online forums were invited to participate in this retrospective epidemiologic study. The inclusion criteria were experience in triathlon sport for at least 12 months and amateur status. Triathlete ability level ranged from novice club to top-level athlete. The study was conducted in accordance to the Declaration of Helsinki and was approved by the local ethics committee of the University of Münster (2016-280-f-S).

After obtaining verbal informed consent from the athletes or their legal guardians, participating athletes were asked to complete an online-based questionnaire, which was created using the program EFS Unipark (QuestBack GmbH, Cologne, Germany). Its content and structure were based on the recent literature, [3, 6, 8–10] Special emphasis was placed on the distinction between the terms “acute” and “overuse” injury. Any event associated with shoulder complaints, due to which the athlete required general or specific treatment (as decided by a treating physician or the athlete themselves) or which caused training modification or pause for at least a day, was defined as an injury. Acute injuries were defined as those caused by a single traumatic event, such as a collision, twist or overstretching. Injuries, which could not be attributed to such an event, but resulted from high amount of repetitive motion sequences and load pattern of the single disciplines of triathlon, were classified as overuse injuries. This complex definition was chosen to reflect the fact that a time-loss definition of injury alone does not adequately capture the majority of overuse injuries in particular.[11]

The questionnaire consisted of three parts. The first part documented the athletes’ competition and training habits, including the weekly training volume in all three disciplines in the past twelve months. In the second part, the athletes reported acute and overuse injuries of the shoulder sustained during the past 12 months. Injured athletes were asked to document when and how the injury was sustained, its influence on training habits, the intensity and duration of resulting complaints, as well as the treatment they received. Preexisting shoulder complaints prior to taking up triathlon were also documented in this section. The last section collected anthropometric data.

A total of 216 questionnaires were returned. 11 respondents were excluded because they did not complete all sections of the questionnaire. 7 athletes had less than 12 months of experience in triathlon, while another 5 respondents were professional athletes and were also excluded, leaving 193 athletes as the subject of this study.

### Statistical analysis

Contingency tables were analyzed using Pearson’s chi-squared test. Continuous variables were checked for normality using the Kolmogorov-Smirnov test. Normally distributed data are presented with their mean values and standard deviations (SD) and were compared with the independent samples t-test. Data without normal distributions are presented with their median values and interquartile ranges (IQR), while non-parametric analyses were performed with the Mann-Whitney U test. Binary logistic regression was used to identify important predictors of injuries.

Statistical analyses were performed with the IBM SPSS Statistics software version 22.0 (IBM Corp., Armonk, NY). All p values are two-sided; a p value < 0.05 was considered significant.

## Results

The characteristics of the participating triathletes are summarized in Table 1. 109 athletes (56%) came from a running background, 40 athletes (21%) from swimming, 25 athletes (13%) from cycling and 19 athletes (10%) from other sports. The average weekly training duration amounted to 10.7 ± 4.7 hours (h)/week (range, 3.3–34). 3.8 ± 2.3 h/week were spent cycling (range, 0.3–16), 3.5 ± 1.7 h/week running (range, 0.3–10), 2.2 ± 1.2 h/week swimming (range, 0–8) and 1.2 ± 1.2 h/week performing weight training (range, 0–6). 28 athletes (15%) had had at least one episode of shoulder complaints before taking up triathlon. 115 athletes (60%) regularly used paddles during swimming training.

**Table 1:** participant characteristics and sex related differences. Normally distributed data are presented with their mean values and SD (in brackets range), data without normal distributions are presented with their median values and IQR.

	**all participants (n = 193)**	**men** **(n = 133)**	**women** **(n = 60)**	**p value**
**age (y)**	33(IQR 27–43)	35(IQR 28–45)	29(IQR 25–36)	0.001
**triathlon experience (y)**	4(IQR 2–9)	5(IQR 3–10)	4(IQR 1–8)	0.026
**weight (kg)**	73 (IQR 67–80)	76 (IQR 71–82)	64 (IQR 59–69)	<0.0001
**height (cm)**	180 ± 8 (157–205)	183 ± 7 (170–205)	172 ± 6 (157–187)	<0.0001
**BMI**	22.4 (IQR 21.4–23.9)	22.8 (IQR 21.8–24.4)	21.4 (IQR 20.3–22.5)	<0.0001
**number of races in the last 12 months**	4 (IQR 2–6)	5 (IQR 2–7)	3.5 (IQR 2–6)	0.060

At least one acute shoulder injury was sustained by 12 participants (6%), while 36 triathletes (19%) experienced at least one overuse injury in the previous 12-month period. 5 triathletes (3%) sustained both an acute and an overuse injury. The acute injury rate amounted to 0.11 per 1000h of training and the overuse injury rate to 0.33 per 1000h of training. 7 acute injuries (58%) occurred during cycling, while 31 overuse injuries (86%) were thought to be primarily associated with swimming. The median reported duration of symptoms following an acute or overuse injury was 2.5 weeks (IQR 1.25–4.0), while the average maximal pain intensity, scored on a visual analog scale, amounted to 3.9 ± 2.3 (range, 1–10).

29 injured athletes (67%) paused at least aspects of their training due to the sustained injuries (Table 2) in order to alleviate the injury-related complaints. The various treatments that the injured athletes received are listed in Table 3. Regarding possible risk factors, there was no association between athletes’ age, height, weight, BMI, or a history of shoulder complaints prior to taking up triathlon and acute or overuse injuries (Table 4). Moreover, the triathlon experience in years was also not associated with acute (p = 0.194) and overuse injuries (p = 0.702). Concerning the influence of sex, male athletes had a trend for sustaining more acute injuries then female athletes (8% vs. 2%, p = 0.079), however there were no significant differences in the rate of overuse injuries (21% vs. 13%, p = 0.203).

**Table 2:** Training modification after sustained injuries. Normally distributed data are presented with their mean values and SD (in brackets range), data without normal distributions are presented with their median values and IQR.

training modification	n (%)	duration (wks)
**pause of all training**	8 (19%)	3 (IQR 2–8)
**pause of swimming**	28 (65%)	5.5 ± 5.9 (1–24)
**pause of cycling**	9 (21%)	6.2 ± 8.5 (1–28)
**pause of running**	7 (16%)	3.9 ± 3.3 (1–9)
**no sport abstention**	14 (33%)	-

**Table 3:** Treatment of sustained injuries.

treatment	n	%
**physical therapy**	14	33
**use of nonsteroidal anti-inflammatory drugs (NSAIDs)**	9	21
**chiropractic treatment**	8	19
**surgical treatment**	2	5
**shoulder injection**	1	2
**acupuncture**	1	2
**no treatment**	14	33

**Table 4:** Possible risk factors for the occurrence of acute and overuse injuries (p values).

	**acute** **injuries**	**overuse injuries**
**sex**	0.109	0.235
**age**	0.282	0.899
**weight**	0.310	0.499
**height**	0.086	0.556
**BMI**	0.983	0.587
**history of shoulder complaints before triathlon sport**	0.531	0.907

The weekly training hours were significantly associated to acute injury rate, with injured athletes reporting a significantly higher amount of weekly training compared to athletes without acute injuries (Table 5). Subgroup analysis revealed that athletes with acute injuries spent a significantly higher amount of time per week doing weight training and had a trend for a higher weekly duration of cycling training, compared to athletes without acute injuries (Table 5). On the other hand there were no significant differences in the weekly running and swimming training hours between athletes with and without acute injuries (Table 5).

**Table 5:** Association between duration of training and acute or overuse injuries (mean values and SD, in brackets range).

	acute injury		p value	overuse injury		p value
	yes	no		yes	no	
**total training duration/week (h)**	15.0 ± 7.4 (7.5–34)	10.4 ± 4.3 (3.3–29)	0.010	11.0 ± 5.1 (3.8–29)	10.6 ± 4.6 (3.3–34)	0.745
**weight training/week (h)**	2.0 ± 1.0 (0–3)	1.2 ± 1.2 (0–6)	0.007	1.4 ± 1.4 (0–6)	1.2 ± 1.2 (0–6)	0.452
**cycling training/week (h)**	5.4 ± 3.9 (1–16)	3.7 ± 2.1 (0.25–14)	0.088	4.0 ± 2.1 (1–10)	3.7 ± 2.4 (0.25–16)	0.302
**running training/week (h)**	4.5 ± 2.6 (1–10)	3.4 ± 1.6 (0.3–10)	0.151	3.3 ± 1.9 (0.3–10)	3.5 ± 1.7 (0.3–10)	0.396
**swimming training/week (h)**	2.9 ± 1.5 (1–5)	2.2 ± 1.2 (0–8)	0.100	2.5 ± 1.2 (0.6–5)	2.2 ± 1.3 (0–8)	0.063

Athletes coming from a cycling background had a significantly higher acute injury rate, compared to athletes who did not have a cycling background (16% vs. 5%, p = 0.030). Further analysis showed that athletes with a cycling background spent significantly more time on cycling training with a mean of 4.9 ± 3.0 h/week (range, 1.5–16), compared to 3.6 ± 2.1 h/week (range, 0.25–14) for other athletes (p = 0.014).

The amount of training time per week was not associated with the rate of overuse injuries (Table 5). Subgroup analysis demonstrated that athletes with overuse injuries had a trend for a higher weekly duration of swimming training compared to athletes without overuse injuries. On the other hand there were no significant differences in the weekly duration of running, cycling and weight training between athletes with and without overuse injuries (Table 5).

There were also no significant differences in the rate of overuse injuries between athletes with a swimming background and athletes with other backgrounds (15% vs. 20%, p = 0.505). Athletes with a swimming background did not spend significantly more time on swimming training, compared to other athletes (p = 0.249), with an average of 2.5 ± 1.4 h/week (range, 0.5–8) compared to an average of 2.2 ± 1.2 h/week (range, 0–5).

We found a significant association between the use of paddles during swimming training and the development of overuse injuries, with 24% of the athletes using paddles sustaining overuse injuries, compared to 10% of the athletes who did not use paddles (p = 0.014). Athletes using paddles spent significantly more time on swimming training compared to athletes who did not use paddles (2.6 ± 1.3 h/week vs. 1.7 ± 1.0 h/week, p<0.0001). However logistic regression identified only paddles (p = 0.038) and not the duration of swimming training (p = 0.428) as a risk factor for the development of overuse injuries.

Finally, we examined the influence of the number of triathlon races the athletes participated in in the last 12 months and found that athletes with acute injuries had a median of 6 races (IQR 3–7.75), compared to 4 races (IQR 2–6) for athletes without acute injuries (p = 0.098). There were no statistical differences in the number of races in the individual distances between athletes with or without acute injuries. On the other hand, athletes with overuse injuries participated in a significantly higher number of races (median, 5.5 races; IQR 3.25–7), compared to athletes without overuse injuries (median, 4 races; IQR 2–6 –p = 0.005). This difference appears to be owed to a significantly higher number of races in the Olympic distance (median, 2 races; IQR 1–4 vs. median, 1 race; IQR 0–2 –p = 0.007) and a trend for a higher number of races in the Sprint distance (median, 2 races; IQR 1–4 vs. median, 2 races; IQR 1–3 –p = 0.069), with no statistical differences in the number of races in the Long and Ironman distance.

## Discussion

Although the shoulder is one of the most common sites of injury identified in triathletes,[1, 3, 7] available studies in the literature have, to our knowledge, only evaluated parameters associated with triathlon-related injuries in general, or focused on other localizations, as the knee and the back.[12, 13] However, in order for preventive measures to be recommended, specific data on both the incidence and possible risk factors for the development of musculoskeletal complaints are needed.[2, 10] We therefore performed this study in order to assess possible parameters associated with acute and overuse injuries of the shoulder in triathletes.

One of the most interesting findings of our study was that the regular use of paddles during swimming training was significantly associated with overuse injuries. Although we are not aware of any studies examining the impact of paddle use during training in triathletes, several analyses of competitive swimmers have also identified the use of paddles as a risk factor for shoulder injuries.[14, 15] A possible reason for this is the expansion of the hand surface area leading to an increased resistance during the catch and pull phase of the stroke, which results in an increased strain on the anatomical structures of the shoulder.[16] Some researchers therefore recommend limiting or even avoiding the use of paddles during training, at least in swimmers returning from an injury or with shoulder complaints.[16, 17]

The athletes using paddles in our study also spent a significantly higher amount of time on swimming training, compared to other athletes. The weekly amount of training time has also been previously proposed as a possible risk factor for the development of shoulder injuries in swimmers.[18, 19] However, logistic regression showed that the amount of time spend on swimming training was not associated with overuse injuries in our study, leaving paddles as a key risk factor which should be addressed in primary or secondary prevention.

Acute injuries of the shoulder were significantly associated with the duration of strength training per week in our cohort. Previous studies examining this factor have produced conflicting results. In a study of 155 triathletes Korkia et al. reported that participation in strength training had no influence on the overall incidence of acute injuries in general.[7] On the other hand, Greipp examined 168 swimmers and found that an increased incidence of shoulder injuries was related to increased intensity and duration of weight training.[20] In another study of 1262 competitive swimmers, McMaster and Troup also demonstrated an association between shoulder pain and weight training, although their study did not clearly distinguish between acute and overuse injuries.[14] Taking our results into consideration, good coaching and supervision of weight training might also be a plausible preventive measure against the development of acute shoulder injuries in triathletes, especially when new exercises are incorporated in the training plan.[21]

Another risk factor for acute shoulder injuries in our analysis was a cycling background. A possible explanation for this is that the majority of acute injuries were accidents occurring during cycling training, and athletes with a cycling background spent significantly more time on cycling training than other athletes. Bike accidents have previously been identified as the reason for the majority of acute injuries in a study of 174 triathletes,[1] while the shoulder is reported to be the most common site of traumatic cycling.[22] Effective strategies to prevent cycling injuries of the shoulder are lacking.[23] With the exception of helmets, other protectors are rarely used in cycling and are not required by cycling clubs. Prevention of falls as a primary prevention goal is also difficult to achieve, especially in the setting of a race.

As with most studies of this kind, one of the limitations of our study is the sampling method used, as injured athletes may be more interested and thus more likely to fill out a questionnaire analyzing triathlon-related injuries than uninjured athletes, leading to a possible selection bias.[7, 10, 13] We attempted to address this problem from the beginning and put emphasis on the goal to identify preventive measures and optimizing training habits, rather than just assessing acute and overuse injuries. Another limitation lies in the retrospective nature of our analysis, introducing the risk of recall bias.[2, 3] However, Gabbe et al. have previously demonstrated in a study assessing the accuracy of a 12 month injury history recall in a population of 70 community level Australian football players that, while recall accuracy declined as the requested level of detail increased, all athletes were able to recall whether or not they were injured during the previous year and approximately 80% of the athletes could accurately recall the number of injuries and body regions injured.[24] The same study did show that the recall accuracy declined to approximately 60%, when athletes were required to simultaneously recall the number of injuries, body region and injury diagnosis.[24] This low accuracy might be attributed to the difficulty for athletes to recollect a specific medical diagnosis, which is why we refrained from evaluating specific diagnoses in our study.

Another possible limitation regarding the subject of recall bias is whether triathletes could adequately distinguish between acute and overuse injuries given the 12-month recall period of our study. While we were unable to identify any studies, which have evaluated this specific issue in particular, it has previously been shown in the literature that the provision of a clear definition of injury can help improve the memory of participants through the provision of specific prompts.[24, 25] For that reason we specifically provided clear and detailed definitions for the terms “acute” and “overuse injuries” to the athletes participating in our study.

Recall bias could obviously also affect the reported load of the triathletes. However, previous studies have reported a reliable level of accuracy for athletes recalling their practice history, possibly because training activities play such an important part in the athletes’ lives that they can recall accurate numbers.[26–28] Another study by Clarsen et al., evaluating overuse injuries that professional cyclists experienced in the previous 12-month period, found no significant differences between exposure estimates and data from accurate training records, although the authors acknowledged that it was impossible to know for certain how accurate these estimates were.[29]

Finally, another limitation of our study regarded the documentation of regular paddle use by the triathletes, which was not quantified. On the other hand, given the retrospective nature of the data collection, we felt that a more detailed quantification might introduce further issues with recall bias, as described above.

## Conclusions

Overuse injuries of the shoulder are much more common in amateur triathletes than acute injuries. The regular use of paddles during swimming training appears to be a risk factor for the development of overuse injuries, while an increased duration of weight and cycling training seems to be associated with a higher rate of acute injuries. Whereas cycling accidents are difficult to address with specific interventions, future studies should examine whether good coaching and supervision of the use of paddles and weight training might lead to a decrease in the rate of acute and overuse injuries of the shoulder in triathletes.

## Supporting information

S1 File Questionnaire used for the study, translated in English. (DOCX)

S2 File Original questionnaire used for the study in German. (DOCX)

## References

[1] Andersen CA, Clarsen B, Johansen TV, Engebretsen L. High prevalence of overuse injury among iron-distance triathletes. Br J Sports Med. 2013;47(13):857–61. doi: 10.1136/bjsports-2013-092397 . 23902775 10.1136/bjsports-2013-092397

[2] Burns J, Keenan AM, Redmond AC. Factors associated with triathlon-related overuse injuries. J Orthop Sports Phys Ther. 2003;33(4):177–84. doi: 10.2519/jospt.2003.33.4.177 . 12723674 10.2519/jospt.2003.33.4.177

[3] Gosling CM, Gabbe BJ, Forbes AB. Triathlon related musculoskeletal injuries: the status of injury prevention knowledge. J Sci Med Sport. 2008;11(4):396–406. doi: 10.1016/j.jsams.2007.07.009 . 17869584 10.1016/j.jsams.2007.07.009

[4] Vleck V, Millet GP, Alves FB. The impact of triathlon training and racing on athletes' general health. Sports Med. 2014;44(12):1659–92. doi: 10.1007/s40279-014-0244-0 . 25292108 10.1007/s40279-014-0244-0 PMC7099231

[5] Main LC, Landers GJ, Grove JR, Dawson B, Goodman C. Training patterns and negative health outcomes in triathlon: longitudinal observations across a full competitive season. J Sports Med Phys Fitness. 2010;50(4):475–85. . 21178935

[6] Gosling CM, Forbes AB, Gabbe BJ. Health professionals' perceptions of musculoskeletal injury and injury risk factors in Australian triathletes: a factor analysis. Phys Ther Sport. 2013;14(4):207–12. doi: 10.1016/j.ptsp.2012.09.004 . 23177357 10.1016/j.ptsp.2012.09.004

[7] Korkia PK, Tunstall-Pedoe DS, Maffulli N. An epidemiological investigation of training and injury patterns in British triathletes. Br J Sports Med. 1994;28(3):191–6. ; PubMed Central PMCID: PMCPMC1332066. 8000820 10.1136/bjsm.28.3.191 PMC1332066

[8] Shaw T, Howat P, Trainor M, Maycock B. Training patterns and sports injuries in triathletes. J Sci Med Sport. 2004;7(4):446–50. . 15712500 10.1016/s1440-2440(04)80262-7

[9] Vleck VE, Bentley DJ, Millet GP, Cochrane T. Triathlon event distance specialization: training and injury effects. J Strength Cond Res. 2010;24(1):30–6. doi: 10.1519/JSC.0b013e3181bd4cc8 . 20042924 10.1519/JSC.0b013e3181bd4cc8

[10] Egermann M, Brocai D, Lill CA, Schmitt H. Analysis of injuries in long-distance triathletes. Int J Sports Med. 2003;24(4):271–6. doi: 10.1055/s-2003-39498 . 12784169 10.1055/s-2003-39498

[11] Clarsen B, Myklebust G, Bahr R. Development and validation of a new method for the registration of overuse injuries in sports injury epidemiology: the Oslo Sports Trauma Research Centre (OSTRC) overuse injury questionnaire. Br J Sports Med. 2013;47(8):495–502. doi: 10.1136/bjsports-2012-091524 . 23038786 10.1136/bjsports-2012-091524

[12] Clements K, Yates B, Curran M. The prevalence of chronic knee injury in triathletes. Br J Sports Med. 1999;33(3):214–6. ; PubMed Central PMCID: PMCPMC1756167. 10378077 10.1136/bjsm.33.3.214 PMC1756167

[13] Manninen JS, Kallinen M. Low back pain and other overuse injuries in a group of Japanese triathletes. Br J Sports Med. 1996;30(2):134–9. ; PubMed Central PMCID: PMCPMC1332377. 8799598 10.1136/bjsm.30.2.134 PMC1332377

[14] McMaster WC, Troup J. A survey of interfering shoulder pain in United States competitive swimmers. Am J Sports Med. 1993;21(1):67–70. doi: 10.1177/036354659302100112 . 8427371 10.1177/036354659302100112

[15] O'Donnell CJ, Bowen J, Fossati J. Identifying and managing shoulder pain in competitive swimmers: how to minimize training flaws and other risks. Phys Sportsmed. 2005;33(9):27–35. doi: 10.3810/psm.2005.09.195 . 20086378 10.3810/psm.2005.09.195

[16] Tovin BJ. Prevention and Treatment of Swimmer's Shoulder. N Am J Sports Phys Ther. 2006;1(4):166–75. ; PubMed Central PMCID: PMCPMC2953356. 21522219 PMC2953356

[17] Johnson JE, Sim FH, Scott SG. Musculoskeletal injuries in competitive swimmers. Mayo Clin Proc. 1987;62(4):289–304. . 3550306 10.1016/s0025-6196(12)61906-5

[18] Sein ML, Walton J, Linklater J, Appleyard R, Kirkbride B, Kuah D, et al Shoulder pain in elite swimmers: primarily due to swim-volume-induced supraspinatus tendinopathy. Br J Sports Med. 2010;44(2):105–13. doi: 10.1136/bjsm.2008.047282 . 18463295 10.1136/bjsm.2008.047282

[19] Tate A, Turner GN, Knab SE, Jorgensen C, Strittmatter A, Michener LA. Risk factors associated with shoulder pain and disability across the lifespan of competitive swimmers. J Athl Train. 2012;47(2):149–58. ; PubMed Central PMCID: PMCPMC3418126. 22488280 10.4085/1062-6050-47.2.149 PMC3418126

[20] Greipp JF. Swimmer's Shoulder: The Influence of Flexibility and Weight Training. Phys Sportsmed. 1985;13(8):92–105. doi: 10.1080/00913847.1985.11708859 . 27442739 10.1080/00913847.1985.11708859

[21] Mazur LJ, Yetman RJ, Risser WL. Weight-training injuries. Common injuries and preventative methods. Sports Med. 1993;16(1):57–63. . 8356377 10.2165/00007256-199316010-00005

[22] Silberman MR. Bicycling injuries. Curr Sports Med Rep. 2013;12(5):337–45. doi: 10.1249/JSR.0b013e3182a4bab7 . 24030309 10.1249/JSR.0b013e3182a4bab7

[23] Sikic M, Mikocka-Walus AA, Gabbe BJ, McDermott FT, Cameron PA. Bicycling injuries and mortality in Victoria, 2001–2006. Med J Aust. 2009;190(7):353–6. . 19351307 10.5694/j.1326-5377.2009.tb02446.x

[24] Gabbe BJ, Finch CF, Bennell KL, Wajswelner H. How valid is a self reported 12 month sports injury history? Br J Sports Med. 2003;37(6):545–7. doi: 10.1136/bjsm.37.6.545 ; PubMed Central PMCID: PMCPMC1724702. 14665599 10.1136/bjsm.37.6.545 PMC1724702

[25] Askling C, Lund H, Saartok T, Thorstensson A. Self-reported hamstring injuries in student-dancers. Scand J Med Sci Sports. 2002;12(4):230–5. . 12199872 10.1034/j.1600-0838.2002.00237.x

[26] Baker J, Cote J, Abernethy B. Sport-Specific Practice and the Development of Expert Decision-Making in Team Ball Sports. Journal of Applied Sport Psychology. 2003;15(1):12–25. doi: 10.1080/10413200305400

[27] Memmert D, Baker J, Bertsch C. Play and practice in the development of sport‐specific creativity in team ball sports. High Ability Studies. 2010;21(1):3–18. doi: 10.1080/13598139.2010.488083

[28] Moesch K, Elbe AM, Hauge ML, Wikman JM. Late specialization: the key to success in centimeters, grams, or seconds (cgs) sports. Scand J Med Sci Sports. 2011;21(6):e282–90. doi: 10.1111/j.1600-0838.2010.01280.x . 21401722 10.1111/j.1600-0838.2010.01280.x

[29] Clarsen B, Krosshaug T, Bahr R. Overuse injuries in professional road cyclists. Am J Sports Med. 2010;38(12):2494–501. doi: 10.1177/0363546510376816 . 20847225 10.1177/0363546510376816

