# Supplementary material for: Risk factors for acute injuries and overuse syndromes of the shoulder in amateur triathletes - A retrospective analysis
Source: PLoS One. 2018 Jun 1;13(6):e0198168. doi: 10.1371/journal.pone.0198168 (PMC5983428; doi:10.1371/journal.pone.0198168)
Supplement: S1 File — (DOCX) [file pone.0198168.s001.docx]

Questionnaire

Dear triathletes!

Triathlon is booming! The time spent on training with sophisticated plans and on competition events is fun. However, musculoskeletal complaints are not uncommon.

That’s what our study is all about. To be more precise: **we are evaluating acute and overuse injuries of the shoulder in amateur triathletes.**

Our goal is to gain an overview of the type and frequency of shoulder injuries and identify possible links with the intensity of training, competition or other factors.

Based on these data we would try to develop recommendations for training plans and possible preventive measures.

Please take part in our anonym survey (approximately 5-10 minutes, regardless of whether you have/had shoulder complaints or not.

We thank your for your cooperation and wish you a successful season!

Part 1:

Training and competion habits in the past 12 months

**How much time on average did you spent on training per week?**

Swimming hours

Cycling hours

Running hours

Weight training hours

**How often did you stretch prior to training?**

(see Appendix)

Swimming never rarely about 50% often always

Cycling never rarely about 50% often always

Running never rarely about 50% often always

**How often did you stretch after training?**

(see Appendix)

Swimming never rarely about 50% often always

Cycling never rarely about 50% often always

Running never rarely about 50% often always

**Please state the number of completed competition events.**

Sprint distance

Olympic distance

Long distance

Ironman distance

Other (Cycling, running race etc. – please indicate the distance in km)

**How many years of triathlon experience do you have?**

years

**Which discipline did you (most) practice prior to triathlon?**

(see Appendix)

Swimming

Cycling

Running

Other, namely:

**Do you regularly practice in other sports?**

No Yes, namely:

**How do you train?**

(multiple answers possible)

In a club

No club membership

With a personal trainer

According to a training plan

**Do you regularly use paddles during swimming training?**

No

Yes

Part 2:

Questions relating to acute and overuse injuries of the shoulder

**Did you have any episodes of shoulder complaints prior to taking up triathlon?**

No

Yes

**Did you have shoulder complaints in the past 12 months?**

No

Yes

**What caused the shoulder complaints? In which discipline?**

(see Appendix)

Accident/acute injury Running Cycling Swimming

Overuse damage Running Cycling Swimming

Other reason, namely:

**Please shortly describe the cause of the complaints in your own words:**

**Did a physician diagnose a specific lesion?**

(see Appendix)

No

Yes, namely:

**Which treatment did you receive?**

(multiple answers possible)

None

Physical therapy

Chirotherapy, osteopathy

Physical measures

Medication

Surgical treatment

Other, namely:

**How long did you abstain from sport because of these shoulder complaints in the past 12 months?**

(see Appendix)

No abstention

Swimming pause in weeks

Cycling pause in weeks

Running pause in weeks

Pause of all training/sport in weeks

**Did you modify your training due to the complaints?**

No

Yes, namely:

**What was the average intensity of your shoulder pain in the past 12 months?**

0 no pain – 10 worst possible pain

**How long did the longest episode of shoulder complaints last in the past 12 months?**

Weeks

Part 3

Anthropometric data

**Sex**

Male

Female

**Are you an amateur or professional triathlete?**

(see Appendix)

Amateur

Professional

**Age**

years

**Height**

cm

**Weight**

kg

**Thank you for participating in this study. Keep having fun with and successes in triathlon and stay healthy!**

Appendix

**How often did you stretch prior to training?**

Please state **rarely**, if you stretched less than half of the time prior to training, and **often**, if you stretched more than half of the time prior to training.

**How often did you stretch after training?**

Please state **rarely**, if you stretched less than half of the time after training, and **often**, if you stretched more than half of the time after training.

**Which discipline did you (most) practice prior to triathlon?**

If you practiced more than one discipline prior to taking up triathlon, please indicate here the discipline you spent the most time with.

**What caused the shoulder complaints? In which discipline?**

If your complaints were due to an injury, please state **accident/acute injury** or **overuse damage**, and indicate the discipline, during which the complaints manifested. An injury is defined as any event, due to which:

- you or your treating physician initiated a general (e.g. anti-inflammatory drugs, massages, physical measures etc.) or a specific treatment (e.g. surgical treatment, specific physical treatment of the injured muscle groups, extracorporeal shockwave treatments etc.) or

- a pause (interruption of the planned training in at least one discipline for at least one day) or a modification (change of the training plan either in one discipline, or between several disciplines) of training was necessary.

The term **accident/acute injury** refers to injuries, which were caused by a single traumatic event, such as collision, twist or overstretching. The term **overuse damage** refers to injuries, which cannot be attributed to such a single traumatic event, but resulted from a high amount of repetitive motion sequences and load patterns of the single disciplines.

If the complaints cannot be attributed to an injury, as defined above, please state **other cause** and shortly describe what was the cause of the complaints.

**Did a physician make a specific diagnosis?**

If you cannot remember the exact diagnosis, please state **yes** and than state **do not recollect**.

**How long did you abstain from sport due to shoulder complaints in the past 12 months?**

Please indicate under **pause of all training/sport in weeks** the number of weeks in which you abstained from all sports (if applicable), and the under the single disciplines the number of weeks you abstained from the planned training in that discipline (if applicable).

**Are you an amateur or professional triathlete?**

Please state **amateur**, if you do not earn or contribute to your livelihood through triathlon; if you do, please state **professional**.
