# Supplementary material for: Risk factors for acute injuries and overuse syndromes of the shoulder in amateur triathletes - A retrospective analysis
Source: PLoS One. 2018 Jun 1;13(6):e0198168. doi: 10.1371/journal.pone.0198168 (PMC5983428; doi:10.1371/journal.pone.0198168)
Supplement: S2 File — (DOCX) [file pone.0198168.s002.docx]

Fragebogen

Liebe Triathletinnen und Triathleten,

Triathlon boomt! Neben all dem Spaß am Training und Wettkampf, ausgefeilten Trainingsplänen und allem was dazugehört, treten aber auch häufig Beschwerden am Bewegungsapparat auf.

Genau das ist das Thema unserer Studie. Genauer noch: **wir untersuchen Akutverletzungs- und Überlastungsschäden an der Schulter bei Amateurtriathleten.**

Ziel ist es, einen Überblick über die Art und Häufigkeit dieser Verletzungen zu gewinnen und mögliche Zusammenhänge mit Trainings- und Wettkampfintensitäten oder anderen Faktoren aufzudecken.

Darauf aufbauend sollen Empfehlungen für die Trainingsplanung, aber auch für mögliche Vorbeugemaßnahmen entwickelt werden.

Bitte nehmen Sie an der anonymen Befragung (ca. 5-10 Minuten) teil, unabhängig davon, ob Sie Beschwerden haben oder hatten.

Vielen Dank für Ihre Mitarbeit und weiterhin eine erfolgreiche Saison!

Teil 1:

Trainingsgewohnheiten und Wettkämpfe der letzten 12 Monate

**Wie war der durchschnittliche Trainingsumfang pro Woche?**

Schwimmen Std

Radfahren Std

Laufen Std

Kraftsport Std

**Wie häufig erfolgte ein Stretching vor dem Training?**

(s. Anhang)

Schwimmen nie selten zu 50% häufig immer

Radfahren nie selten zu 50% häufig immer

Laufen nie selten zu 50% häufig immer

**Wie häufig erfolgte ein Stretching nach dem Training?**

(s. Anhang)

Schwimmen nie selten zu 50% häufig immer

Radfahren nie selten zu 50% häufig immer

Laufen nie selten zu 50% häufig immer

**Bitte geben sie die Anzahl der absolvierten Wettkämpfe an**.

Sprint/Volksdistanz

Kurzdistanz/Olympische Distanz

Mitteldistanz

Langdistanz/Ironman

Sonstige (Radrennen, Wettlauf etc. mit km-Angabe)

**Seit wie vielen Jahren betreiben Sie Triathlonsport?**

Jahre

**Von welcher Disziplin sind Sie (am ehesten) zum Triathlon gekommen?**

(s. Anhang)

Schwimmen

Rennrad

Laufen

Sonstige, nämlich:

**Üben sie regelmäßig noch andere Sportarten aus?**

Nein Ja, nämlich:

**In welchem Rahmen trainieren Sie?**

(Mehrfachantworten möglich)

Im Verein

Ohne Vereinsbindung

Mit eigenem Trainer

Nach Trainingsplan

**Benutzen Sie regelmäßig Paddles beim Schwimmtraining?**

Nein

Ja

Teil 2:

Schulterspezifische Fragen zu Verletzungen und Überlastungen

**Bestanden bei Ihnen schon vor der Aufnahme des Triathlonsports Beschwerden in der Schulterregion?**

Nein

Ja

**Hatten Sie in den letzten 12 Monaten Beschwerden in der Schulter?**

Nein

Ja

**Was war für die Schulterbeschwerden ursächlich? In welcher Disziplin?**

(s. Anhang)

Unfall/plötzliches Ereignis Laufen Rad Schwimmen

Überlastung Laufen Rad Schwimmen

Andere Ursache und zwar:

**Bitte beschreiben Sie die Ursache der Beschwerden kurz mit eigenen Worten:**

**Wurde eine Diagnose von einem Arzt gestellt?**

(s. Anhang)

Nein

Ja, nämlich folgende

**Welche Therapie erfolgte?**

(Mehrfachantworten möglich)

Keine

Krankengymnastik

Chirotherapie, Osteopathie

Physikalische Maßnahmen

Medikamente

Operation

Sonstige Therapie, nämlich:

**Wie lange haben Sie aufgrund Ihrer Schulterbeschwerden in den letzten 12 Monaten mit dem Sport pausiert?**

(s. Anhang)

Gar nicht

Schwimmen Pause in Wochen

Rad Pause in Wochen

Laufen Pause in Wochen

Komplette Sportpause in Wochen

**Haben Sie aufgrund der Beschwerden Ihr Training umgestellt?**

Nein

Ja und zwar wie folgt:

**Wie stark waren Ihre Schulterschmerzen im Durchschnitt in den letzten 12 Monaten?**

0 keine Schmerzen – 10 stärkster vorstellbarer Schmerz

**Wie lange hielt die längste Schmerzepisode in den letzten 12 Monaten an?**

Wochen

Teil 3:

Anthropometrische Daten

**Geschlecht**

Mann

Frau

**Sind Sie Amateur oder Profi?**

(s. Anhang)

Amateur

Profi

**Alter**

Jahre

**Größe**

cm

**Gewicht**

Kg

**Vielen Dank für Ihre Teilnahme und weiterhin viel Spaß, Erfolg und Gesundheit beim Triathlon!**

Anhang

**Wie häufig erfolgte ein Stretching vor dem Training?**

Geben Sie bitte **selten** ein, wenn Sie vor weniger als die Hälfte der Trainingseinheiten ein Aufwärmen/Stretching durchgeführt haben, und **häufig**, wenn Sie vor mehr als die Hälfte der Trainingseinheiten ein Aufwärmen/Stretching durchgeführt haben.

**Wie häufig erfolgte ein Stretching nach dem Training?**

Geben Sie bitte **selten** ein, wenn Sie nach weniger als die Hälfte der Trainingseinheiten ein Aufwärmen/Stretching durchgeführt haben, und **häufig**, wenn Sie nach mehr als die Hälfte der Trainingseinheiten ein Aufwärmen/Stretching durchgeführt haben.

**Von welcher Disziplin sind Sie (am ehesten) zum Triathlon gekommen?**

Falls Sie vor Aufnahme des Triathlonsports mehr als eine Sportart betrieben haben, geben Sie bitte hier die Disziplin ein, bei der Sie vor Aufnahme des Triathlons die meiste Zeit verbracht haben.

**Was war für die Schulterbeschwerden ursächlich? In welcher Disziplin?**

Wenn Ihre Beschwerden an eine Verletzung zurückzuführen sind, geben Sie bitte **Unfall/plötzliches Ereignis** oder **Überlastung** ein, sowie die Disziplin, bei der die Verletzung aufgetreten ist. Mit Verletzung sind alle Ereignisse gemeint, aufgrund deren:

- Sie oder Ihr behandelnder Arzt eine allgemeine (z.B. Endzündungshemmer, Massagen, physikalische Maßnahmen usw.) oder spezifische Therapie (z.B. Operation, spezifische Beübungen der verletzten Muskelgruppen, Stoßwellentherapie etc.) eingeleitet haben, oder

- eine Trainingspause (Unterbrechung des geplanten Trainings in mindesten einer Disziplin für mindestens einen Tag), oder eine Trainingsmodifizierung (Veränderung des Trainingsplans entweder in einer Disziplin oder zwischen verschiedenen Disziplinen) erfolgte.

Mit **Unfall/plötzliches Ereignis** sind Verletzungen gemeint, die durch ein einzelnes traumatisches Ereignis, wie z.B. eine Kollision, eine Verdrehung oder eine Überdehnung bedingt sind. Mit **Überlastung** sind Verletzungen gemeint, die nicht auf ein solches einzelnes traumatisches Ereignis zurückgeführt werden können, aber als Ergebnis einer hohen Anzahl von sich wiederholenden Bewegungsabläufen und Belastungsmustern in den einzelnen Disziplinen gemeint.

Wenn die Beschwerden nicht an eine Verletzung, wie oben definiert, zurückzuführen sind, geben Sie bitte **andere Ursache** an und schildern Sie bitte kurz, was zu den Beschwerden geführt hat.

**Wurde eine Diagnose von einem Arzt gestellt?**

Wenn Sie sich an die genaue Diagnose nicht erinnern können, geben Sie bitte **ja** und dann als Freitext **nicht erinnerlich** ein.

**Wie lange haben Sie aufgrund Ihrer Schulterbeschwerden in den letzten 12 Monaten mit dem Sport pausiert?**

Geben Sie bitte unter **komplette Sportpause in Wochen** die Anzahl der Wochen ein, in denen Sie absolut kein Sport betrieben haben (falls zutreffend), und dann bei den einzelnen Disziplinen die Anzahl der Wochen ein, in denen Sie in dieser Disziplin außerplanmäßig nicht trainiert haben (falls zutreffend).

**Sind Sie Amateur- oder Profi?**

Geben Sie bitte **Amateur** ein, wenn sie nicht Ihr Lebensunterhalt durch den Triathlonsport finanzieren, sonst geben Sie bitte **Profi** ein.
